# Interallied Surgical Conference, 4th Session (Conclusions)

**Published:** 1918-08

**Authors:** 


### ABSTRACTS

### SURGERY

**Interallied Surgical Conference, 4th Session,** *La Presse Médicale,* April 11, 1918.

The Interallied Surgical Conference for the Study of War

Wounds held its meeting at the Val-de-Grâce, March 11-16. The seven following questions were discussed :

1. *Blood Transfusion.* Indication and technique;

2. Diagnosis, physiology, pathology and treatment of trench foot (frozen feet);

3. Treatment of wounds of the pelvis (especially rectum and bladder);

4. Pseudarthrosis consecutive to war wounds;

5. Conservative operations in the treatment of foot injuries by war projectiles;

6. Osteosynthesis in the treatment of war fractures;

7. Analysis of methods of wound sterilization.

After having heard and discussed the reports presented on these different questions, the Conference adopted the following conclusions :

### I. *Conclusions on Blood Transfusion.*

Blood transfusion gives results sufficiently definite to be considered the method of choice in the treatment of severe hemorrhages.

I. *Indications.* a) *During the first hours after the wound :* Indications are observed by means of clinical study. They can be shown more clearly by frequent taking of blood pressure and by counting the number of the red blood corpuscles, which give indications in wounds of the limbs.

In the advanced posts and when circumstances make such examinations impossible, the indications will be furnished solely by the *clinical symptoms.*

In cases of circulatory collapse resulting from a superacute affection (gas gangrene), transfusion has not given favorable results.

In shock, the indications for transfusion are not sufficiently definite.

b) *During treatment.* Post-hemorrhagic globular anemia is not usually serious. It does not justifiy transfusion, if the general condition of the patient is satisfactory.

II. *Preliminary Precautions.* a) It is useless to examine and classify the donors to avoid transmission of diseases such as syphilis and malaria.

b) Fatal accidents have been observed in cases where the globules of the donor were agglutinated by the patient's plasma. These accidents are very unusual. They can surely be avoided by a very easy agglutination test. It is therefore absolutely indispensable to look for agglutination in all cases, as far as possible, and to classify the givers accordingly.

In war work, transfusion may be made in advanced posts and in the H. O. E. (*Field Hospitals*) In the latter, all these precautions must be taken. In the advanced posts, transfusion can be done, even if it is impossible to make the agglutination test, since the danger is relatively small.

c) Transfusion must not be undertaken except with an apparatus in perfect condition and one which can be thoroughly sterilized.

III. *Technic.* The method must correspond to the quantity of blood transfused. Gratifying experiments have been made in the use of blood preserved during many days. This method will be of great benefit, especially in advanced posts during intensive military activity.

The *indirect* methods of transfusion of fresh blood are more easily employed than the vasculary anastomosis; *citrated* blood is used in the former, aspiration of pure blood in paraffined ampoules or in syringes in the latter. The three methods have given good results.

As a rule, transfusion must be made as soon as possible after the injury. Nevertheless, it cannot be performed before the hemorrhage has stopped. In hemorrhages of the chest or abdomen, or in wounds of limbs, transfusion should be performed before or during the operation, according to the patient's condition.

### II. *Conclusions on Trench Feet.*

1. Trench foot is a pathologic condition caused by damp cold, often complicated by secondary infection.

2. Its evolution has the four following periods (Raymond).

   1. Period of painful anesthesia;
   2. Edema;
   3. Blisters;
   4. Scabs.

Three clinical forms can be described.

a) A mild form (85 to 90 o/o of cases) marked by painful anesthesia, edema and redness; b) A fairly severe form (13 to 14 o/o of cases) marked by blisters and limited scabs; c) A severe form (1 o/o of cases on the average) marked by an increase of gangrenous phenomena and the appearance of septic accidents. This form mutilates severely and causes death.

3. Trench foot, especially in the severe forms, is very often complicated with tetanus and gas gangrene. It is also liable to relapse and recur. Trench foot occurs nearly exclusively on soldiers staying in trenches, particularly in certain trenches. Soldiers from warm countries, the negroes especially, are more often victims than the Europeans. In Italy, the soldiers from the South have suffered more than those from the North. Adolescence, hyperhydrosis, and a previous attack are predisposing causes.

4. Blood stasis caused by standing for a long time, long immobility and defective position (half kneeling), compression of the leg and poor venous circulation, as produced by spiral puttees, and especially a long stay in cold dampness (muddy, flooded trenches, shell holes), constitute the principle causes of trench feet.

6. Trench foot can be confused with chilblain and frozen foot. Congelation is marked by early massive mortification of part of a limb (fore foot, entire foot, etc.); the trench foot, on the contrary, by a limited destruction (sphacelous patches on the dorsum, sole, and toes progressively extending to other tissues of the foot). The first condition is observed in intense dry colds, especially in the mountains; the second is produced only by damp weather, at low altitudes (valleys, plains). The trench foot disappears when it freezes.

Chilblains are characterized, at the beginning at least, by severe itching, whereas trench foot, in its light forms, is marked by a painful anesthesia without any itching. Diagnosis between ulcerated chilblains and the ulcerated phlyctenas of trench foot, may often be difficult.

6. The treatment of trench foot is preventive and curative. The preventive treatment, energetically applied and supervised, prevents the occurrence of trench foot, or at least makes it very unusual. It includes :

a) Collective individual measures (cleaning, greasing, and massage of the feet; change of socks in the shelter; proper adjustment of the spiral puttees and of all that can produce compression of the leg). Our Belgian confreres ascribe the extremely low incidence of trench foot in their army to the suppression of spiral puttees.

### *Curative Treatment.*

a) *Slight Forms.* Every second or third day, lukewarm foot bath and use of borated-camphorated soap; daily dressing of the foot with a moist borated-camphorated dressing.

b) *Severe Forms.* If only blisters are present, they should be opened and camphorated ether, with a moist borated-camphorated dressing, applied. If scabs are present continue the same treatment patiently. Scabs must not be taken off surgically but just scarified, without bleeding, so that the modifying agents can act on the underlying tissues. Wait for spontaneous elimination, carefully watch complications and treat them surgically and open widely from the beginning. The operation must, as a rule, be deferred.

In every case, this treatment must be supplemented by a preventive antitetanic treatment (injection of antitetanic serum every eight days, until healing of the wounds).

### III. *Conclusions on Wounds of the Pelvis, Especially of the Rectum and Bladder.*

I. *Simple Wounds of the Pelvis.* Treatment depends on the general therapeutics of war wounds of the soft parts or bones. Comminutive fractures of the ilium, particularly, require wide trephining.

The extraction of projectiles (splinters are often driven in the psoas iliacus) is very difficult and must be done systematically.

II. *Wounds of the Bladder* : (a) *Intraperitoneal* : Justifiy a laparotomy and suture; (b) *Extra-peritoneal* : Super-pubic wounds can be treated by primitive vesical suture.

The wounds of the walls and base of the bladder, which cannot be reached by operation do not require an immediate systematic cystostomy. The surgical treatment of the entrance wound and of its direction insure sufficient drainage.

III. *Wounds of the Rectum* : (a) *Intraperitoneal* : Like all intestinal wounds, these justify laparotomy with suture. (b) *Extra peritoneal* : In the majority of cases treatment consist in the flattening of the wound, followed by plugging of the rectal wound.

Constipation of the patient is an indispensable adjuvant.

In splintering with extensive separation, the fixing of the rectum, completed if necessary by a posterior rectotomy, will be the treatment of choice. Primitive colostomy has very exceptional indications.

IV. *Associated Wounds of the Bladder and Rectum.* The majority of these heal up after the surgical treatment of the extravesical direction of the projectile. Colostomy should be reserved for very large vesicorectal openings.

Primitive cystotomy is very often useless. The retention sound, ventral decubitus, micturition in the genu pectoral position favor spontaneous healing of the vesicorectal fistula.

IV. *Conclusions on Treatment of Pseudarthroses* :

I. The inevitable primary cause of pseudarthroses is the *primitive* destruction of a part of the diaphysis. The other actual causes — infection, careless extraction of splinters (esquillectomy) and bad reductions should be avoided.

A certain number of pseudarthroses may be prevented by sterilizing the focus of the fracture, conservative esquillectomy, good reduction, carefully watched, and, in some well defined cases, immediate or early osteosynthesis.

II. Except in exceptional cases, pseudarthroses must be operated on *late*, when the skin wound has completely healed and when clinically the inflammatory reaction is over.

The late and lesser inflammations must be detected by such methods as active and passive movements, elastic bands, and intensive massage.

III. From a therapeutic standpoint two cases must be recognized.

(a) In the simple pseudarthroses and in certain others associated with loss of substance in segments of limbs having only one bone, after refreshing the edges of the bones according to pathological anatomy, metallic osteosynthesis can be performed. The best method appears to be the fixation by metallic plate with screws — the screws being driven as far as possible from the center of the pseudarthroses.

The combination of the metallic prothesis and of the osteoperiostic graft has given very good results.

(b) A pseudarthrosis with loss of bone substance necessitates in most cases the bone or osteoperiostic graft.

IV. A perfect asepsis and a complete excision of the fibrous tissue surrounding the fragments and of certain diseased bone portions are the indispensable conditions of success.

V. *Conclusions on the Results of Conservative Operations in Wounds of the Foot by War Projectiles.*

Since the integrity of the sole of the foot is very desirable, only the strictly necessary incisions and resections should be performed in that region. Healing by first intention must be looked for in every case. The same thing must be looked for on the dorsum of the foot when the cicatrix retraction might hamper the movements of the sole of the foot. It is even permissible to resect certain bones so as to favor the primitive and secondary reunion of the wound and the normal position of the plantar teguments.

The amputation of one or many toes causes little difficulty of movement. The conservation of a single toe, especially the first or the fifth, is very often embarrassing.

The disarticulation of the metatarsals with conservation of the corresponding toes generally gives unfavorable results.

The resection of the first and fifth toes with their metatarsals also gives defective results.

When the three metatarsals, II, III, IV, have heen resected, they give to the foot a pointed form which seriously impedes walking and standing.

Generally, the disappearance of three metatarsals hampers greatly the statics of the foot.

The transmetatarsal amputation, with a good plantar flap, gives a very favorable result, whether made at the anterior or at the farthest posterior part of the metatarsus.

Following Lisfranc's disarticulation, walking is easy and even flexible, if the rest of the foot is in good condition. This difficult operation can be simplified by leaving the metatarsal cases in the wound. The pre-scaphoido-cuboidian amputation produces good functional results.

Summing up, all the operations on the anterior tarsus give favorable results if there is no complication due to the scar or to the condition of the overlying articulations.

Chopart's amputation, when performed under good conditions and well attented to, can give a good result; but equinismus and the prolapse of the stump often cause functional troubles which make it *inferior* to Lisfranc's and Syme's operations. Partial astragalo-calcanean or horizontal resections of the calcaneum prevent a tendency to equinism.

The sub-astragalian amputation, Pirogoff's amputation, and especially Syme's, allow easy and quick walking.

Operations on the posterior tarsus are, on the contrary, very often followed by functional troubles. Total or sub-total astragalostomy gives good results, but they are less favorable than in peace time; infections, articular, and tendinous stiffness, inadequate after-care of the foot position, are the causes of this deficiency.

The total, or nearly total, ablation of the calcaneum, if not followed by bone regeneration, produces serious troubles, with very frequent tibia-tarsian or mediotarsian ankylosis.

Partial resections, posterior or inferior, give less favorable results if the foot is kept at right angles for the total duration of treatment.

Associated resection of the calcaneum and the astragalum gives generally bad results.

*Atypical Operations.* The resections of the anterior tarsus, scaphoid, cuboid, are often accompanied by equinism with valgus or varus or falling of the arch. An orthopedic shoe greatly helps movements. The ablation of either of these two bones seems to be equally serious.

The results of atypical operations on many bones of the anterior tarsus depend on the state of conservation of the arch of the foot, on the value of the plantar supports, and on the movements of the articulations and tendons, much more than on the localization of the operation itself.

The faulty attitudes of the foot, with free articulations, can be cured or ameliorated by sections or by tendinous transplantations. Transplantations will be particularly useful when certain tendons have disappeared.

Certain faulty attitudes with ankylosis necessitate secondary operations on the skeleton (cuneiform resection, astragalectomy).

As a rule, conservative operations on the metatarsus give excellent results; but on the posterior tarsus, ablations of the calcaneum or the combined resection of many bones, often cause more severe functional troubles than those resulting from disarticulation or Syme's operation.

*Notation.* Professor Depage notes that in articular suppuration of the tarsus long persisting, even after astragalectomy, the anterior and inward inversion of the foot by large sections of tendons and ligaments if kept bandaged in this position, favors the disinfection of the focus. The foot can be restored to its normal position after 8 or 15 days.

### VI. *Conclusions on Osteosynthesis in War Fractures.* (Pseudarthroses excepted).

We must consider the primitive osteosynthesis, and the osteosynthesis during the infection period of the fracture.

*Primitive Osteosynthesis.* The possibility of applying to a great number of wounds, complicated with fractures the primitive immediate or deferred suture, justifies the practice of immediate osteosynthesis.

The indications of this osteosynthesis are few.

1. Certain articular fractures in which osteosynthesis appears to be the method of choice for the return to the anatomical and functional state.

2. The irreducible diaphysis fractures, or those impossible of maintaining correct reduction (specially sub-condylar fractures of the femur, fractures of the fore-arm, etc.) and the presence of large splinters off their axis.

However, the perfection of modern apparatus allows, in most cases, a satisfactory correction without osteosynthesis.

Primitive osteosynthesis is a difficult operation which can bring about serious complications. It must be performed only by specialists, and its indications are few. In the British Army, primitive osteosynthesis is very seldom performed, and this for two reasons: 1st, the good results of modern apparatus; 2nd, the bad results of osteosynthesis in the past.

*Osteosynthesis During the Infection Period.* Osteosynthesis during the infection period is accepted by some, but formally rejected by others.

The former say that it lessens the infection of the fracture focus, is not accompanied by osteomyelitis of long duration, and very seldom is the cause of secondary sequestra.

As a whole, the results are favorable.

The indications are the impossibility of reduction, or keeping correctly reduced some diaphyseal fracture of the limbs.

However, the perfection of modern apparatus gives such satisfactory reductions that osteosynthesis is very seldom indicated.

The majority is in favor of the temporary osteosynthesis by the screwed plate.

VII. (This Section Was Not Summarized.)

